# A Critical Time-Window for the Selective Induction of Hippocampal Memory Consolidation by a Brief Episode of Slow-Wave Sleep

**DOI:** 10.1007/s12264-018-0303-x

**Published:** 2018-11-09

**Authors:** Yi Lu, Zheng-Gang Zhu, Qing-Qing Ma, Yun-Ting Su, Yong Han, Xiaodong Wang, Shumin Duan, Yan-Qin Yu

**Affiliations:** Department of Neurobiology, Institute of Neuroscience, National Health Commission and Chinese Academy of Medical Sciences Key Laboratory of Medical Neurobiology, Zhejiang University School of Medicine, Hangzhou, 310058 China

**Keywords:** Parafacial zone, Slow-wave sleep, Memory consolidation, Hippocampus, Optogenetics

## Abstract

Although extensively studied, the exact role of sleep in learning and memory is still not very clear. Sleep deprivation has been most frequently used to explore the effects of sleep on learning and memory, but the results from such studies are inevitably complicated by concurrent stress and distress. Furthermore, it is not clear whether there is a strict time-window between sleep and memory consolidation. In the present study we were able to induce time-locked slow-wave sleep (SWS) in mice by optogenetically stimulating GABAergic neurons in the parafacial zone (PZ), providing a direct approach to analyze the influences of SWS on learning and memory with precise time-windows. We found that SWS induced by light for 30 min immediately or 15 min after the training phase of the object-in-place task significantly prolonged the memory from 30 min to 6 h. However, induction of SWS 30 min after the training phase did not improve memory, suggesting a critical time-window between the induction of a brief episode of SWS and learning for memory consolidation. Application of a gentle touch to the mice during light stimulation to prevent SWS induction also failed to improve memory, indicating the specific role of SWS, but not the activation of PZ GABAergic neurons itself, in memory consolidation. Similar influences of light-induced SWS on memory consolidation also occurred for Y-maze spatial memory and contextual fear memory, but not for cued fear memory. SWS induction immediately before the test phase had no effect on memory performance, indicating that SWS does not affect memory retrieval. Thus, by induction of a brief-episode SWS we have revealed a critical time window for the consolidation of hippocampus-dependent memory.

## Introduction

Sleep is conserved across species from *Caenorhabditis elegans* to humans, suggesting that it is critical to survival [1–3]. Sleep has been associated with many functions, particularly learning and memory [1, 2, 4, 5]. However, despite extensive studies, its role in memory processing is still controversial and elusive [5, 6].

As a fundamental ability, memory formation comprises three major processes: encoding, consolidation, and retrieval. Different sleep patterns have been reported to affect different memory processes [2]. It has been suggested that slow-wave sleep (SWS) preferentially supports hippocampus-dependent declarative memory, while rapid eye movement (REM) sleep benefits non-declarative aspects of memory, such as procedural, implicit, and emotional memory [7]. Studies on the roles of different sleep patterns on memory have usually been done by comparing the effect of early retention sleep, which is dominated by SWS, with late retention sleep, which is dominated by REM sleep [8, 9]. However, early retention sleep also includes REM sleep, and late retention sleep also includes SWS sleep. Some knowledge about the effect of sleep on memory has come from sleep deprivation [10, 11], which induces stress, and this may secondarily interfere with learning and memory. Furthermore, it is also impossible to selectively eliminate SWS and leave REM sleep undisturbed.

The parafacial zone (PZ) has been reported to be a SWS-promoting center [12, 13]. In the present study, we found that optogenetic stimulation of GABAergic neurons in the PZ immediately induced SWS in vesicular GABA transporter – channelrhodopsin 2 – enhanced yellow fluorescent protein (VGAT-ChR2-EYFP) mice. Taking advantage of this approach, we were able to precisely control the timing of SWS in these mice and investigate, with high temporal precision, the effects of induced SWS on memory processes.

## Materials and Methods

### Animals

All experimental procedures were approved by the Zhejiang University Animal Experimentation Committee. Adult (8 weeks old) C57BL/6J and VGAT-ChR2-EYFP transgenic male mice were used. Before experiments started, animals were held in individual chambers for at least 5 days. The temperature (22 °C–23 °C), humidity (40%–60%), and circadian rhythm (12 h light/dark cycles, starting at 07:00) were maintained constant in custom-designed stainless-steel cabinets. Food and water were available *ad libitum*.

### Stereotaxic Surgery

All VGAT-ChR2-EYFP mice were anesthetized with pentobarbital sodium (100 mg/kg, i.p.) and mounted on a small-animal stereotaxic frame (Stoelting Corp., Wood Dale, IL). Then an optical fiber (AniLab, Ningbo, Zhejiang, China) was unilaterally implanted above the PZ (anteroposterior, − 5.5 mm; mediolateral, 1.4 mm; dorsoventral, − 4.2 mm) and a custom-made electroencephalographic (EEG) and electromyographic (EMG) unit was attached to the skull. EEG signals were recorded from electrodes on the frontal cortices (anteroposterior, 2 mm; mediolateral, 1 mm). Two stainless-steel wires were inserted into neck muscles as EMG electrodes.

### Recording and Analyses of EEG and EMG

After the surgical procedures, mice recovered in individual chambers for at least 1 week. Each animal was transferred to the recording chamber and connected to an EEG/EMG head-stage and an optical fiber. The data cable was connected to a slip-ring device (CFS-22) to allow a mouse to freely move in its cage without tangling the cable. The torsion in the optical fiber was released by an optical commutator (Doric Lenses, Quebec, Canada). The animals were habituated for at least 3 days before EEG and EMG recording.

The EEG and EMG signals from the implanted electrodes were amplified, filtered (EEG, 0.5 Hz–100 Hz; EMG, 10 Hz–500 Hz) using differential AC amplifiers (Model 1700, A-M Systems, Carlsborg, WA), digitized at 200 Hz using PowerLab (ML795, ADInstruments, Dunedin, New Zealand), and recorded using LabChart software (ADInstruments).

The SleepSign program (Kissei Comtec, Matsumoto City, Japan) was used to spectrally analyze the digitally-filtered signals by fast Fourier transformation. The total delta or theta power was represented as the overall power in the spectrum of 0.5 Hz–4 Hz or 4 Hz–10 Hz in a 0.5 Hz–35 Hz window with 0.38 Hz resolution, respectively. The NeuroExplorer (Nex Technology, Littleton, MA) was used to analyze the EEG power spectral density. To normalize the data, the relative EEG power was represented by the ratio of the power spectral density in the different frequency ranges to the average value of total power in the same epoch. To analyze the changes in the EEG spectrum during the transitions among SWS, REM sleep, and wakefulness, we analyzed the EEG power in wakefulness or REM sleep during the 30-min photostimulation [14].

Sleep state was scored with the SleepSign software. All scoring was automatic on the basis of the signatures of the EEG and EMG waveforms in 4-s epochs. Wakefulness was defined as desynchronized EEG and heightened tonic EMG activity with phasic bursts; SWS as synchronized, high-amplitude, low-frequency (0.5 Hz–4 Hz) EEG and greatly reduced EMG activity; and REM sleep as having a pronounced theta rhythm (4 Hz–10 Hz) and a flat EMG (atonia). All classifications of states assigned by SleepSign were examined visually and corrected manually.

### *In Vivo* Photostimulation

An optical fiber was inserted into the unilateral cannula 1 h before the behavioral experiments. The light pulse-trains (473 nm, 40 Hz/5 ms) for VGAT-ChR2-EYFP mice were programmed using a stimulator (PG 4000A, Cygnus Technology, Inc., Delaware Water Gap, PA); the same was done for the control group but the stimulator was not switched on. Experiments were carried out from 19:00 to 07:00 in the active period. The light-induced SWS in the active period was measured by offline scoring of the EEG/EMG recordings.

### Immunohistochemistry

For immunohistochemistry, adult mice were deeply anesthetized with pentobarbital sodium (100 mg/kg, i.p.) and transcardially perfused with normal saline followed by 4% paraformaldehyde in 0.1 mol/L phosphate buffer. The brain was then removed, post-fixed for 4 h, cryoprotected in 30% sucrose, and sectioned coronally at 30 μm on a freezing microtome (CM 1950; Leica, Buffalo Grove, IL). After rinsing with 0.5% Triton-X in 0.1 mol/L PBS for 30 min and blocking with 10% normal bovine serum for 1 h at room temperature, sections were incubated with primary glutamate decarboxylase (GAD) 67 antibody (mouse anti-GAD67, 1:200, Millipore MAB5406) in 0.1 mol/L PBS for 12 h at 4 °C. Sections were then rinsed and incubated for 2 h with Cy3-conjugated donkey anti-mouse secondary antibody (1:1000, Jackson ImmunoResearch, West Grove, PA) at room temperature. Nuclei were stained with 4, 6-diamidino-2-phenylindole (DAPI). Finally, the immunostained sections were analyzed immediately after the sections were rinsed in 90% glycerol and coverslipped.

### Behavioral Testing

#### Pretraining

After being handled for 7 days, the animals were habituated to the arena without stimuli for 10 min–15 min daily for 4 days before commencement of the behavioral testing. All behavioral tests were carried out in the active period (19:00–07:00).

#### Object-in-Place Task

This task consisted of an acquisition (sample) phase and a test phase separated by a 1-h or 6-h delay. In the sample phase, the mice were shown 4 different objects, which were placed in the corners of the arena 15 cm from the walls. Each mouse was placed in the center of the arena and allowed to explore the objects for 5 min. During the delay period, all the objects were cleaned with alcohol to remove any sawdust or olfactory cues. In the test phase, the positions of two of the objects, both on one side of the arena, were exchanged and the mouse was allowed to explore the objects for 5 min. The time spent exploring the two objects that had changed position (unfamiliar objects) was compared with the time spent exploring the two objects that had remained in the same position (familiar objects). The discrimination ratio was the time spent exploring unfamiliar or familiar objects divided by the total time. The two objects were selected randomly for exchange, but they were kept on the same side. If the novel object-in-place recognition (NOR) memory is intact, the mouse spends more time exploring the unfamiliar objects than the familiar objects [15].

#### Y-Maze

The Y-maze apparatus was made of gray polyvinyl chloride with 3 symmetrical arms (30×10×15 cm^3^) without extra- or intra-maze spatial cues, and was evenly illuminated (30 lux). During the first trial (sample phase; 10 min), each mouse was allowed to explore 2 of the 3 arms with the third arm blocked. After a 1-h or 6-h inter-trial interval, each mouse was placed in the center of the Y-maze and allowed to explore all arms freely (test phase; 10 min). An arm entry was counted when all 4 limbs of the mouse entered an arm. The percentage of time spent in the novel arm and the 2 familiar arms was calculated, with a higher preference for the novel arm being defined as intact spatial recognition memory [16].

#### Contextual and Cued Fear Memory

For conditioning, mice were placed in the conditioning chamber, and the room light was switched off. Three minutes later, a 20-s tone (conditioned stimulus; 90 dB, 5 kHz sine wave, 50-ms rising and falling time) was presented and co-terminated with a scrambled 2-s electric footshock (unconditioned stimulus; 0.5 mA). Then, the conditioning procedure was repeated twice at inter-tone intervals of 100 s. Mice were returned to their home-cages 60 s after the last footshock.

To measure the freezing response to the context, 6 h after 3-min training, mice were placed in the same context as the training, and the room light was switched off. One hour later, mice were placed in a different context and the light was also switched off. Three minutes later, a 3-min tone was presented (5 kHz; 90 dB) and the freezing response to the cue (tone) was measured. Mice were returned to their home-cages 60 s after the end of tone presentation. The freezing times during the 3 min in the different contexts were calculated separately.

### Statistical Analysis

Data are presented as mean ± SEM. Student’s *t*-test was used for statistical analysis. In all cases, *P* < 0.05 was taken as the level of significance.

## Results

### Effects of Light-Induced SWS on Memory Consolidation in the Object-in-Place Task

**Fig. 1:**
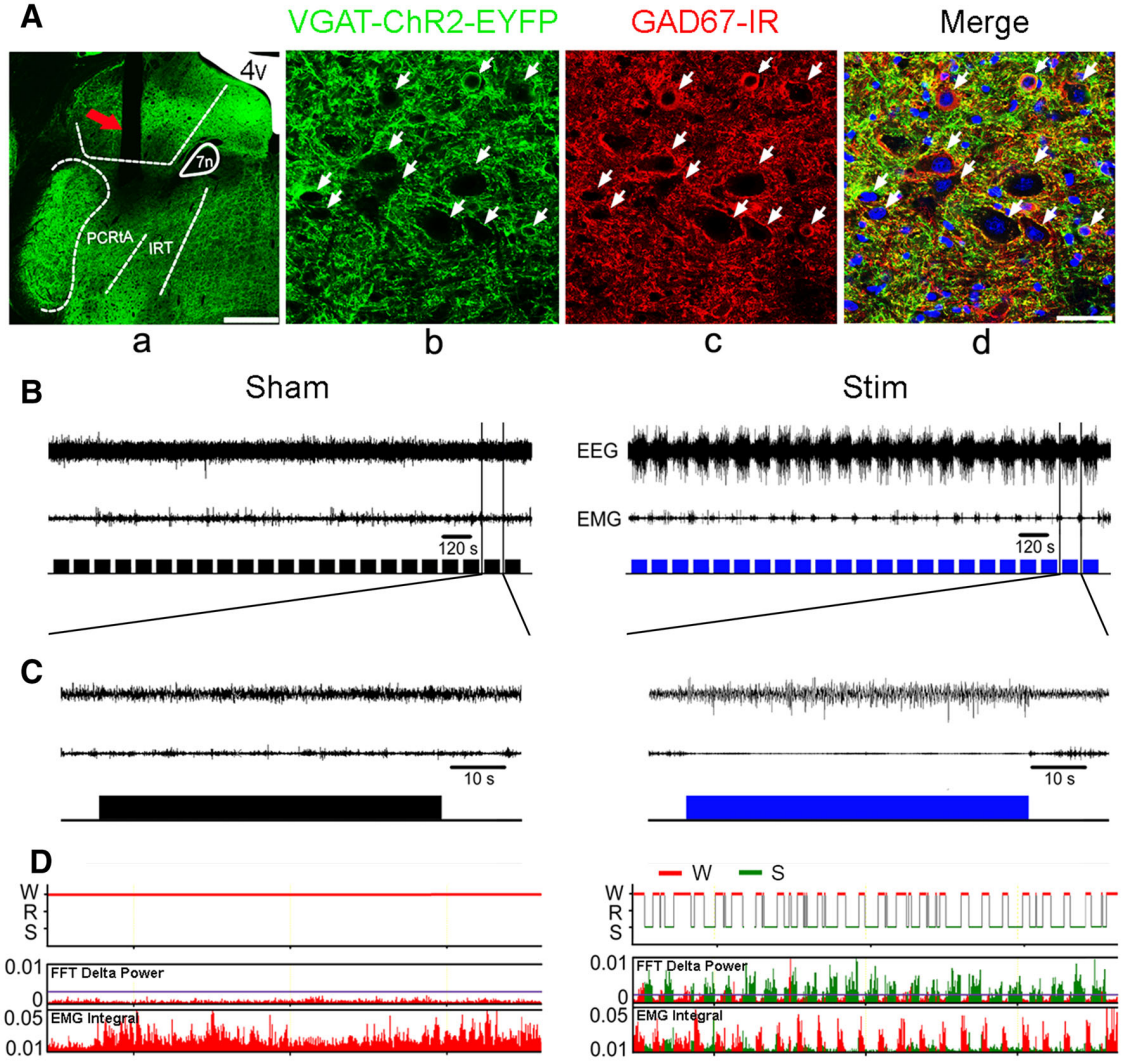
Expression and activation of ChR2 in the PZ of VGAT-ChR2-EYFP mice. **A a**. Representative photomicrograph of the location of an optical fiber. Arrow, track of the fiber. The PZ is composed of the parvocellular reticular nucleus, alpha part (PCRtA) and the intermediate reticular nucleus (IRt). 7n, facial nerve or its root; 4V, 4th ventricle. Scale bar, 500 μm. **b, c, d.** Co-localization (arrows) of ChR2-EYFP and GAD67 immunofluorescence in the PZ neurons of VGAT-ChR2-EYFP mice. Green, ChR2-EYFP; red, GAD67; blue, DAPI. Scale bar, 40 μm. **B** Example recordings of EEG (upper traces) and EMG (middle traces) activity in response to sham (left panel, lower traces) or light (right panel, lower blue traces) stimulation (473 nm, 40 Hz/5 ms) of GABAergic PZ neurons. Note that episodes of typical SWS, as judged by EEG and EMG traces, were induced in an awake mouse by light stimulation, but not by sham stimulation. **C** Example traces from (**B**) showing a typical episode of SWS induced by a train of light stimulation (right panel), but not by sham stimulation (left panel). Upper, middle, and lower traces are EEG, EMG, and pulses of light stimulation, respectively. **D** Fast Fourier transform (FFT)-derived delta (0.5 Hz–4 Hz) power over 0.5 h of sham stimulation (left panel) or light stimulation (right panel, 473 nm, 40 Hz/5 ms) of GABAergic PZ neurons. Red, wakefulness (W); green, SWS (S).

To confirm that the expression of ChR2 was restricted to GABAergic neurons, we carried out immunohistochemical detection of GAD67 in the PZ of VGAT-ChR2-EYFP transgenic mice (Fig. 1A). The results showed that GAD67-positive neurons were co-localized with almost all ChR2-EYFP neurons in the PZ (Fig. 1A).

We found that light stimulated the GABAergic PZ neurons in VGAT-ChR2-EYFP transgenic mice (blue light at 473 nm: pulse width 5 ms, 40 Hz for 60 s, repeated at 20-s intervals), and this instantly and reliably induced pure SWS from wakefulness with an increase of the delta power in the EEG and a decrease of muscle tone as shown in the EMG (Fig. 1B–D).

**Fig. 2:**
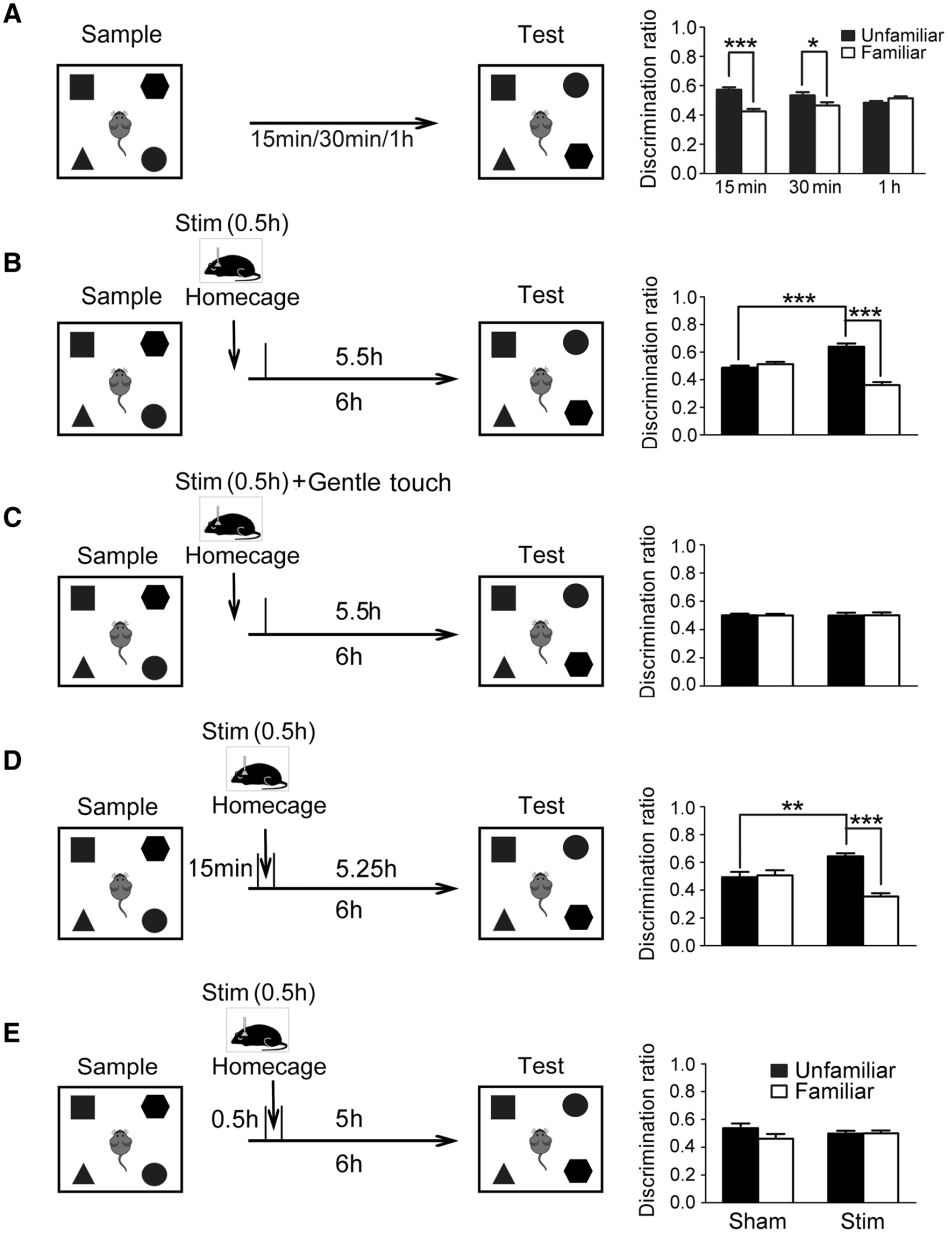
Light-induced SWS enhanced memory consolidation in the object-in-place task. **A** Left panel: diagrams of experimental design and delay intervals of the object-in-place task. Right panel: discrimination ratios of the time mice spent on unfamiliar or familiar objects with intervals of 15 min (*n* = 13), 30 min (*n* = 9), and 1 h (*n* = 13) between the sample and test phases. *P < 0.05, ***P < 0.001 compared with control. **B**–**E** Left panels: diagrams of experimental design of the object-in-place task. Stim, application of light stimulation for 30 min immediately (**B, C**), 15 min (**D**), or 30 min (**E**) after the sample phase. Right panels: discrimination ratios of the time mice spent on unfamiliar or familiar objects with sham (**B**. *n* = 7, **C**. *n* = 5, **D**. *n* = 8, **E**. *n* = 9) or light stimulation (**B**. *n* = 7, **C**. *n* = 6, **D**. *n* = 7, **E**. *n* = 7). **P < 0.01, ***P < 0.001 compared with control.

We then investigated the appropriate interval between the sample phase and the test phase in the NOR task under our experimental conditions. We found that with an interval of 15 min or 30 min between the sample phase and the test phase, the discrimination ratio of the unfamiliar objects increased significantly compared with that of the familiar objects (15 min: 0.57 ± 0.06 *vs* 0.43 ± 0.06, *P* < 0.001, *n* = 13; 30 min: 0.53 ± 0.07 *vs* 0.46 ± 0.07, *P* < 0.05, *n* = 9; Fig. 2A). However, with an interval of 1 h between the sample and test phases, the discrimination ratio of the unfamiliar objects was similar to that of the familiar objects (0.48 ± 0.04 *vs* 0.52 ± 0.04, *P* > 0.05, *n* = 13, Fig. 2A). These results indicated that NOR memory was maintained for < 1 h after the sample phase.

We then photostimulated the GABAergic PZ neurons for 0.5 h immediately after the sample phase and were surprised to find that the NOR memory was still maintained when tested even 6 h after the sample phase. As shown in Fig. 2B, 6 h after the sample phase, the discrimination ratio of the familiar objects was 0.36 ± 0.02 and that of the unfamiliar objects was 0.64 ± 0.02 in the photostimulation group (*P* < 0.001, *n* = 7), in contrast to the control group, in which the discrimination ratios of the familiar and unfamiliar objects were 0.51 ± 0.02 and 0.49 ± 0.02, respectively (*P* > 0.05, *n* = 7). The discrimination ratio of the unfamiliar objects in the photostimulation group was significantly higher than that in control group (*P* < 0.001, *n* = 7, Fig. 2B).

To ensure that the memory consolidation was caused by the light-induced SWS, but not by excitation of the PZ neurons itself, the mice were kept awake by applying a soft tactile stimulus during the photostimulation of GABAergic PZ neurons. The discrimination ratios of the familiar and unfamiliar objects in the control group (0.50 ± 0.01 and 0.50 ± 0.01, respectively, *P* > 0.05, *n* = 5) were similar to those in the photostimulation with gentle touch group (0.50 ± 0.02 and 0.50 ± 0.02, respectively, *P* > 0.05, *n* = 6) (Fig. 2C). This result suggested that the memory was prolonged specifically by the light-induced SWS, but not the activation of PZ GABAergic neurons itself.

Taking advantage of the time-locked SWS induction, we next explored the precise temporal relationship between SWS and memory consolidation. We found that when photostimulation of the GABAergic PZ neurons was delivered 15 min after the sample phase for 30 min, the discrimination ratios of the familiar and unfamiliar objects were 0.36 ± 0.02 and 0.64 ± 0.02 when tested 6 h after the sample phase (*P* < 0.001, *n* = 7, Fig. 2D), whereas the discrimination ratios of the familiar and unfamiliar objects in the control group with sham stimulation were 0.51 ± 0.04 and 0.49 ± 0.04, respectively (*P* > 0.05, *n* = 8, Fig. 2D). The discrimination ratio of the unfamiliar objects in the photostimulation group was significantly higher than that in control group (*P* < 0.01, *n* = 7, Fig. 2D), indicating that the memory was still maintained for 6 h when 30 min of SWS was induced 15 min after the sample phase. However, when the photostimulation-induced SWS was delivered 30 min after the sample phase, the discrimination ratios of the familiar and unfamiliar objects were 0.50 ± 0.02 and 0.50 ± 0.02, assessed 6 h after the sample phase (*P* > 0.05, *n* = 7, Fig. 2E), not significantly different from the control group with sham stimulation. The discrimination ratios of the familiar and unfamiliar objects in the control group with sham stimulation were 0.46 ± 0.03 and 0.54 ± 0.03 (*P* > 0.05, *n* = 9, Fig. 2E). These results indicated that SWS enhances memory with a critical time window of 15 min after learning.

### Light-Induced SWS Enhanced Memory Consolidation in the Y-Maze Test and Contextual Fear Memory

To further characterize the properties of the memory consolidation induced by SWS, we investigated the influence of light-induced sleep on the Y-maze and fear conditioning tasks.

**Fig. 3:**
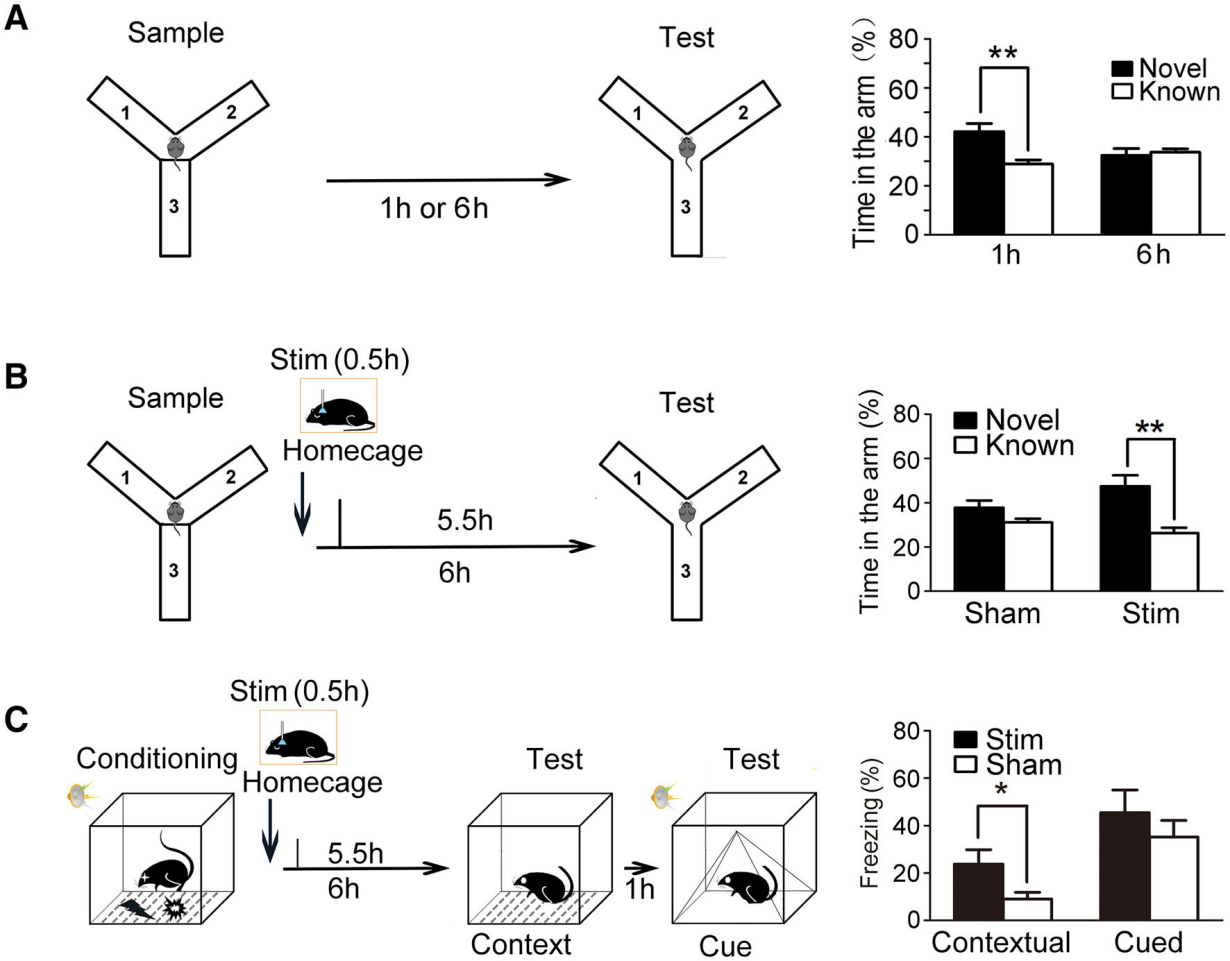
Light-induced SWS enhanced Y-maze spatial memory and contextual fear memory consolidation. **A** Left panel: diagrams of experimental design and the delay intervals of the Y-maze test. Right panel: percentage of time spent in the novel or known arm with intervals of 1 h (*n* = 9) or 6 h (*n* = 10) between the sample and test phases. **P < 0.01 compared with control. **B** Left panel: diagram of experimental design of the Y-maze test. Right panel: percentage of time spent in the novel or known arm with sham (*n* = 9) or light stimulation (*n* = 8). **C** Left panel: diagram of experimental design of the contextual and cued fear memory tests. Right panel: percentage of freezing time with sham (*n* = 13) or light stimulation (*n* = 12) in the contextual and cued fear memory tests. *P < 0.05, **P < 0.01 compared with control.

When the control group was examined 1-h after the sample test, the percentage of time spent in the novel arm (42.16% ± 3.37%) was significantly higher than that spent in each of the known arms (28.92% ± 1.68%) (*P* < 0.01, *n* = 9, Fig. 3A). However, when the control group was examined after a 6-h inter-trial interval, the percentage of time spent in the novel arm (32.41% ± 2.8%) was not significantly different from that spent in each known arm (33.79% ± 1.4%) (*P* > 0.05, *n* = 10, Fig. 3A). Thus, a 6-h inter-trial interval between the sample and test phases of the Y-maze test was selected to determine whether 30 min of SWS consolidated the Y-maze spatial memory. We found that when 30 min of SWS was induced immediately after the sample test, the percentage of time spent in the novel arm (47.45% ± 4.89%) was significantly higher than that spent in the known arm (26.27% ± 2.45%) measured 6 h after the sample test (*P* < 0.01, *n* = 8, Fig. 3B), whereas in the control group with sham stimulation, the percentage of time spent in the novel arm (37.76% ± 3.28%) did not significantly differ from that spent in each known arm (31.12% ± 1.64%, *P* > 0.05, *n* = 9, Fig. 3B).

In the fear memory experiments, mice were placed in the conditioning chamber for conditioning, suffering a scrambled electric footshock 2 s in duration along with a 20-s tone (90 dB). The same photostimulation (0.5 h) was delivered to the mice immediately after conditioning; the context-mediated memory (same context without foot-shock and tone) was tested 6 h later, and the cue-mediated memory (different contexts with the same tone) was tested 7 h later (Fig. 3C). In the contextual fear memory test, the percentage of freezing time with photo-stimulation was 23.69% ± 6.17% (*n* = 12) and with sham stimulation was 9.03% ± 2.83% (*n* = 13) (*P* < 0.05, Fig. 3C). In the cued fear memory test, the percentage of freezing time with the photo-stimulation was 45.45% ± 9.60% (*n* = 12) and with sham stimulation was 35.16% ± 7.00% (*n* = 13) (*P* > 0.05, Fig. 3C).

Taken together, these data suggest that light-induced SWS immediately after learning enhances the consolidation of hippocampus-dependent memory including NOR memory, Y-maze spatial memory, and contextual fear memory, but not cued fear memory.

### Light-Induced SWS Has No Effect on Memory Retrieval

**Fig. 4:**
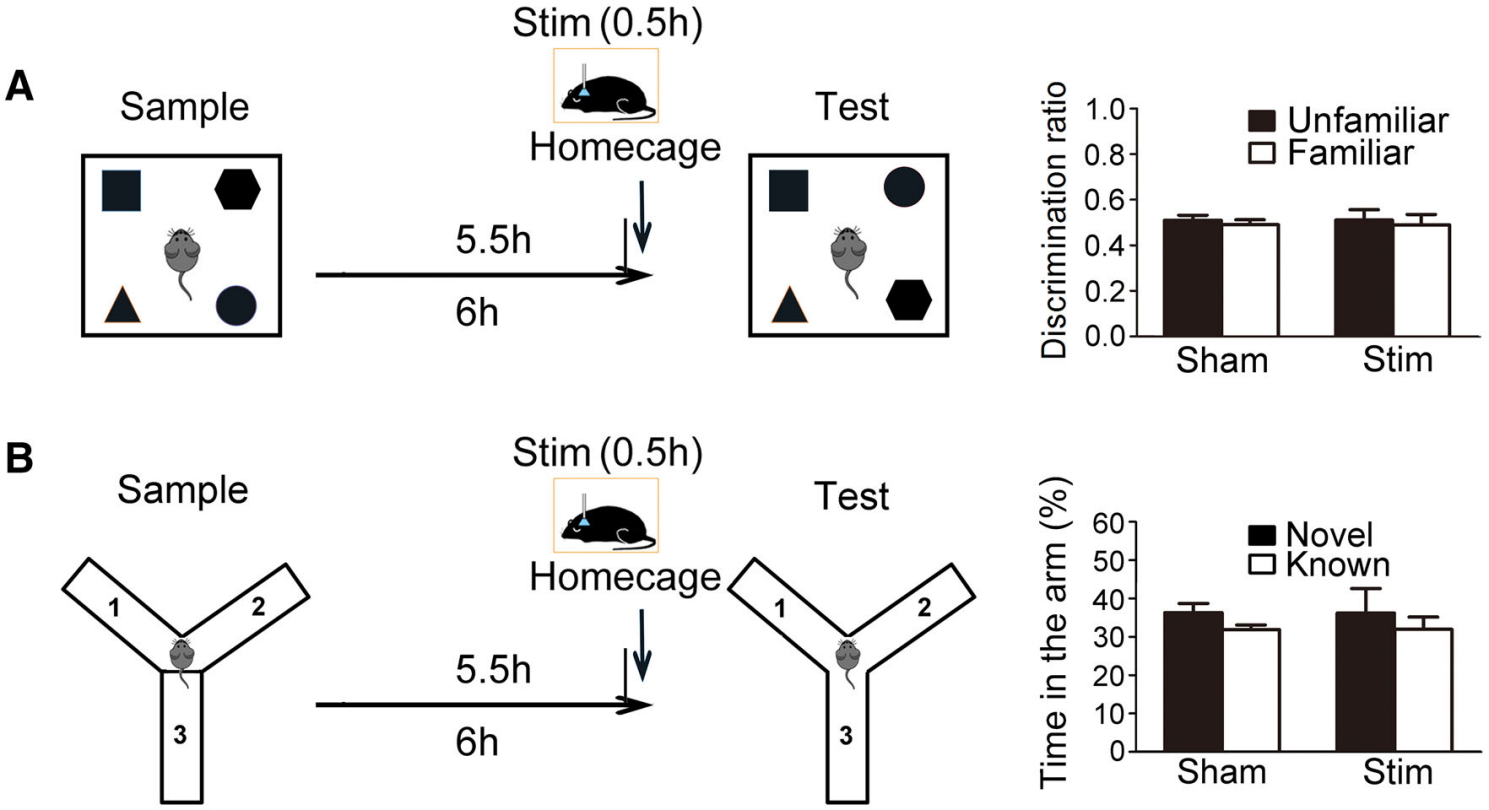
Effect of light-induced SWS on memory retrieval. **A** Left panel: diagrams of the experimental design of the object-in-place task. Right panel: discrimination ratios for time spent on unfamiliar or familiar objects with sham (*n* = 8) or light stimulation (*n* = 7). **B** Left panel: diagram of the experimental design of the Y-maze test. Right panel: percentage of time spent in the novel or known arm with sham (*n* = 9) or light stimulation (*n* = 7).

To investigate the effect of sleep on memory retrieval, we induced SWS for 30 min before the test phase in the object-in-place and Y-maze tests with a 6-h delay between the sample and test phases (Fig. 4). In the object-in-place task, the discrimination ratios of the unfamiliar and familiar objects were 0.51 ± 0.02 and 0.49 ± 0.02, respectively, in the control group with sham stimulation (*P* > 0.05, *n* = 8, Fig. 4A). The discrimination ratios of the unfamiliar and familiar objects were 0.51 ± 0.05 and 0.49 ± 0.05 (*P* > 0.05, *n* = 7, Fig. 4A) when a 30-min SWS induction was applied 30 min before the test phase. The discrimination ratio of the unfamiliar objects in the photostimulation group was not significantly different from that in the control group (*P* > 0.05, Fig. 4A).

In the Y-maze test, the percentages of time spent in the novel and known arms were 36.15% ± 6.42% and 31.92% ± 3.21%, respectively, when a 30-min SWS was induced before the test phase (*P* > 0.05, *n* = 7, Fig. 4B), which were not significantly different from the percentage of time spent in the novel (36.27% ± 2.42%) and known arms (31.87% ± 1.21%) in the control group with sham stimulation (*P* > 0.05, *n* = 9, Fig. 4B).

These results showed that SWS induction immediately before the test phase had no effect on memory retrieval.

## Discussion

It has been suggested that declarative memory initially encoded in the hippocampus is unstable and vulnerable to interference by newly-encoded information. The new memory needs to be gradually stabilized through consolidation processes so that it can be transferred and integrated into pre-existing long-term memory in the neocortex [2, 17].

Sleep was initially thought to play a passive role in memory consolidation by simply protecting new memories from interfering stimuli. However, current studies suggest that sleep provides active off-line processing for system consolidation by the repeated reactivation of new memories, which can then be integrated into pre-existing long-term memories. Indeed, the spatiotemporal patterns of neuronal firing recorded in the hippocampus during exploration of a novel environment or performance of a spatial task are re-played in the same order during subsequent sleep, particularly during SWS [18–22], and this is accompanied by hippocampal sharp wave-ripples [19, 20, 23, 24]. We noted that sharp wave-ripples were also frequently recorded during light-induced SWS (data not shown).

Memory has been divided into short-term memory (STM) that lasts minutes to 1–3 h and long-term memory (LTM) that lasts several hours to days, weeks, or even longer [25–27]. It has been shown that the transformation of STM to LTM requires protein and RNA synthesis [2]. With precisely-controlled SWS, we were able to demonstrate that a brief episode of SWS as short as 30 min was sufficient to prolong NOR memory from 30 min to 6 h, suggesting that SWS plays an active role in transforming STM to LTM. We also found that the SWS-induced transformation of STM to LTM had a critical time-window of 15 min following the training phase. It will be interesting to further explore whether the SWS-induced consolidation of NOR memory is dependent on protein and RNA synthesis. Whether SWS episodes longer than 30 min have a broader time-window for the induction of NOR memory consolidation also requires further study.

Interestingly, we found that 30 min of SWS applied immediately after fear conditioning enhanced context-mediated, but not cue-mediated fear memory. Recognition memory in the NOR test [15, 28], spatial memory in the Y-maze test [16, 29, 30], and contextual fear memory [31–33] are all associated with the hippocampus. The present findings are consistent with the idea that SWS specifically affects hippocampus-dependent memory. Sleep deprivation has been reported to impair memory retrieval [34]. However, in the present study we found that induction of 30 min of SWS immediately before the test phase failed to improve memory in either the NOR or the Y-maze test, suggesting that SWS does not have a direct effect on memory retrieval.

## References

[1] Siegel JM (2009). Sleep viewed as a state of adaptive inactivity. Nat Rev Neurosci.

[2] Rasch B, Born J (2013). About sleep’s role in memory. Physiol Rev.

[3] Pan Y (2016). Sandman is a sleep switch in *Drosophila*. Neurosci Bull.

[4] He C, Hu Z (2017). Homeostasis of synapses: expansion during wakefulness, contraction during sleep. Neurosci Bull.

[5] Sara SJ (2017). Sleep to remember. J Neurosci.

[6] Poe GR (2017). Sleep is for forgetting. J Neurosci.

[7] Maquet P (2001). The role of sleep in learning and memory. Science.

[8] Plihal W, Born J (1997). Effects of early and late nocturnal sleep on declarative and procedural memory. J Cogn Neurosci.

[9] Plihal W, Born J (1999). Effects of early and late nocturnal sleep on priming and spatial memory. Psychophysiology.

[10] Chowdhury A, Chandra R, Jha SK (2011). Total sleep deprivation impairs the encoding of trace-conditioned memory in the rat. Neurobiol Learn Mem.

[11] Yoo SS, Hu PT, Gujar N, Jolesz FA, Walker MP (2007). A deficit in the ability to form new human memories without sleep. Nat Neurosci.

[12] Anaclet C, Lin JS, Vetrivelan R, Krenzer M, Vong L, Fuller PM (2012). Identification and characterization of a sleep-active cell group in the rostral medullary brainstem. J Neurosci.

[13] Anaclet C, Ferrari L, Arrigoni E, Bass CE, Saper CB, Lu J (2014). The GABAergic parafacial zone is a medullary slow wave sleep-promoting center. Nat Neurosci.

[14] Han Y, Shi YF, Xi W, Zhou R, Tan ZB, Wang H (2014). Selective activation of cholinergic basal forebrain neurons induces immediate sleep-wake transitions. Curr Biol.

[15] Barker GR, Warburton EC (2011). When is the hippocampus involved in recognition memory?. J Neurosci.

[16] Wang XD, Rammes G, Kraev I, Wolf M, Liebl C, Scharf SH (2011). Forebrain CRF(1) modulates early-life stress-programmed cognitive deficits. J Neurosci.

[17] Ni B, Wu R, Yu T, Zhu H, Li Y, Liu Z (2017). Role of the hippocampus in distinct memory traces: timing of match and mismatch enhancement revealed by intracranial recording. Neurosci Bull.

[18] Pavlides C, Winson J (1989). Influences of hippocampal place cell firing in the awake state on the activity of these cells during subsequent sleep episodes. J Neurosci.

[19] Wilson MA, McNaughton BL (1994). Reactivation of hippocampal ensemble memories during sleep. Science.

[20] Nadasdy Z, Hirase H, Czurko A, Csicsvari J, Buzsaki G (1999). Replay and time compression of recurring spike sequences in the hippocampus. J Neurosci.

[21] Ji D, Wilson MA (2007). Coordinated memory replay in the visual cortex and hippocampus during sleep. Nat Neurosci.

[22] Ribeiro S, Gervasoni D, Soares ES, Zhou Y, Lin SC, Pantoja J (2004). Long-lasting novelty-induced neuronal reverberation during slow-wave sleep in multiple forebrain areas. PLoS Biol.

[23] Buzsaki G (1989). Two-stage model of memory trace formation: a role for “noisy” brain states. Neuroscience.

[24] Peyrache A, Khamassi M, Benchenane K, Wiener SI, Battaglia FP (2009). Replay of rule-learning related neural patterns in the prefrontal cortex during sleep. Nat Neurosci.

[25] McGaugh JL (1966). Time-dependent processes in memory storage. Science.

[26] Kandel ER (2001). The molecular biology of memory storage: a dialogue between genes and synapses. Science.

[27] Kelleher RJ, Govindarajan A, Jung HY, Kang H, Tonegawa S (2004). Translational control by MAPK signaling in long-term synaptic plasticity and memory. Cell.

[28] Brown MW, Aggleton JP (2001). Recognition memory: what are the roles of the perirhinal cortex and hippocampus?. Nat Rev Neurosci.

[29] Sterlemann V, Rammes G, Wolf M, Liebl C, Ganea K, Muller MB (2010). Chronic social stress during adolescence induces cognitive impairment in aged mice. Hippocampus.

[30] Conrad CD, Galea LA, Kuroda Y, McEwen BS (1996). Chronic stress impairs rat spatial memory on the Y maze, and this effect is blocked by tianeptine pretreatment. Behav Neurosci.

[31] Phillips RG, LeDoux JE (1992). Differential contribution of amygdala and hippocampus to cued and contextual fear conditioning. Behav Neurosci.

[32] Ramamoorthi K, Fropf R, Belfort GM, Fitzmaurice HL, McKinney RM, Neve RL (2011). Npas4 regulates a transcriptional program in CA3 required for contextual memory formation. Science.

[33] Izquierdo I, Furini CR, Myskiw JC (2016). Fear memory. Physiol Rev.

[34] Rahman A, Languille S, Lamberty Y, Babiloni C, Perret M, Bordet R (2013). Sleep deprivation impairs spatial retrieval but not spatial learning in the non-human primate grey mouse lemur. PLoS One.

